# Mothers' Views and Experiences of Digital Maternal Presence Intervention and Skin‐to‐Skin Contact for Neonatal Pain Relief: A Qualitative Study

**DOI:** 10.1002/nop2.70577

**Published:** 2026-04-29

**Authors:** Anna‐Kaija Palomaa, Eeva Talus, Sirpa Keskitalo‐Leskinen, Tarja Pölkki

**Affiliations:** ^1^ Research Unit of Health Sciences and Technology, Faculty of Medicine University of Oulu Oulu Finland; ^2^ Medical Research Center Oulu, Oulu University Hospital University of Oulu Oulu Finland; ^3^ Pediatric and Neonatal Intensive Care Unit Oulu University Hospital Oulu Finland

**Keywords:** critical care nursing, newborn, pain, parents, qualitative research

## Abstract

**Aim:**

To explore mothers' views and experiences on using digital maternal presence intervention (DMPI) and skin‐to‐skin contact (SSC) for pain relief in a neonatal intensive care unit.

**Design:**

A qualitative descriptive study design.

**Methods:**

Purposive sampling was used to recruit 25 mothers whose newborns were treated in a neonatal intensive care unit in Finland and who had participated in a study exploring the effectiveness of non‐pharmacological pain relief methods. Semi‐structured interviews were conducted between May 2023 and May 2024 and analysed using content analysis.

**Results:**

Mothers experienced DMPI as a meaningful way to participate in their newborn's pain care and as a method that supported mother–infant attachment. Although their views on its analgesic effectiveness varied, they perceived DMPI as a womb‐simulating way to comfort their newborns when they could not be present. SSC was generally viewed as effective for pain relief and beneficial for emotional well‐being and bonding; however, mothers described varied feelings about its use during painful procedures and identified challenges that hindered its implementation.

**Conclusion:**

Mothers perceived DMPI as a meaningful way to engage in their newborn's pain relief when they could not be present, whereas SSC was viewed as an effective and emotionally supportive method despite constraints on its use.

**Implications for Practice:**

Maternal participation in pain relief can be supported in neonatal intensive care units by facilitating the use of SSC when feasible and offering DMPI when mothers cannot be present.

**Patient Contribution:**

Mothers of newborns participated in the pre‐testing of the interview framework and in the interviews.

**Reporting Method:**

Consolidated Criteria for Reporting Qualitative Research (COREQ).

**Trial Registration:**

ClinicalTrials.gov NCT04967118

## 1 Introduction

Newborns requiring intensive care undergo repeated painful procedures during hospitalization (Bueno et al. 2024). Repeated exposure to pain, combined with separation from the mother, is a potent stressor that can negatively impact the infant's long‐term development (Filippa et al. 2019). Based on these findings, methods have been developed that incorporate parental presence and involvement in pain relief as an integral part of neonatal pain management (Ullsten et al. 2024). In a neonatal intensive care unit (NICU), care is guided by the principles of a family‐centred model of care (FCC). A key element of this model is the partnership between families and healthcare providers when planning, implementing and making decisions about newborn care (Mikkelsen and Frederiksen 2011). Therefore, understanding mothers' experiences with parent‐driven methods of pain relief for their newborns is essential.

### 1.1 Background

Previous studies on neonatal pain relief have examined the attitudes and perceptions of parents and healthcare providers towards neonatal pain management (Gates et al. 2018; Treiman‐Kiveste et al. 2022), the facilitators and barriers to parental involvement in neonatal pain management (Feng et al. 2025), and the effectiveness of parent‐driven pain relief (Eissler et al. 2022). Research indicates that parents want to be involved in their newborn's pain management in the NICU (Ullsten et al. 2021), and they can use different non‐pharmacological methods to comfort their newborns during painful procedures (Treiman‐Kiveste et al. 2023).

Skin‐to‐skin contact (SSC), in which a diapered newborn is held against a parent's bare chest, is the most extensively studied method of pain relief in which parents actively participate (Ullsten et al. 2024). There is strong evidence that SSC is an effective method for relieving pain during needle‐related procedures (Johnston et al. 2017). Although SSC has been shown to have many positive effects on newborns, including developmental benefits and positive effects on the mother‐infant relationship (Johnston et al. 2017), several quantitative studies indicate that parents report using SSC relatively infrequently for neonatal pain relief (Bujalka et al. 2023; Pölkki et al. 2018; Treiman‐Kiveste et al. 2023). The nature of the procedure (Ullsten et al. 2021) or care culture may limit SSC use as a pain relief method (Treiman‐Kiveste et al. 2023). Parental experience may also influence the use of pain relief methods; parents sometimes report feelings of anxiety or emotional distress related to pain management (Feng et al. 2025).

Digital parent‐driven methods that do not require the parent's physical presence can also be used to relieve neonatal pain (Ullsten et al. 2024). Some of the first auditory stimuli for a foetus are the sounds heard in the womb, such as the mother's voice and heartbeat (Di Fiore et al. 2024). Studies have demonstrated the effectiveness of using intrauterine sounds (Yarahmadi et al. 2024) and a robot that mimics key aspects of skin‐to‐skin contact (Holsti et al. 2019) in alleviating newborn pain. Furthermore, a recently published systematic review and meta‐analysis revealed that playing recorded maternal sounds can significantly increase the comfort level of preterm infants during painful procedures (Li et al. 2023).

Currently, only a few studies have been conducted on how parents perceive the use of specific methods to manage their newborns' pain. A database search revealed two relevant studies. One study examined the experiences of mothers who used the intervention of facilitated tucking to manage their preterm infants' pain (Axelin et al. 2010). The other study investigated parents' perceptions of using a combination of SSC, live parental singing and breastfeeding to relieve their infant's pain (Carlsen Misic et al. 2024). Additionally, Hughes et al. (2024) evaluated the effectiveness of an eHealth website by administering an open‐ended survey regarding experiences with breastfeeding and SSC as methods of pain relief for healthy newborns. To the best of our knowledge, no previous studies have examined the views of parents using SSC or interventions for digital parental involvement in neonatal pain management in the NICU. Understanding mothers' perspectives is crucial for developing family‐centred pain management practices that are feasible, acceptable and supportive within the NICU environment.

## 2 Method

### 2.1 Study Design and Theoretical Framework

The aim of this study was to explore the mothers' views and experiences of using digital maternal presence intervention (DMPI) and skin‐to‐skin contact (SSC) for neonatal pain relief in the NICU. A qualitative descriptive design was adopted to gain a deeper understanding of these perspectives (Willis et al. 2016). The study's theoretical framework was based on the FCC (Larocque et al. 2021). However, inductive content analysis was used for the analysis, meaning no theoretical framework guided it (Elo et al. 2014). This article adheres to the COREQ checklist (Appendix S1) for reporting qualitative studies (Tong et al. 2007).

### 2.2 Study Setting and Participants

Participants were recruited by the first author from a Level III NICU (Barfield et al. 2012) in Finland between May 2023 and May 2024. The unit has 12 beds for level III neonatal care and eight beds for specialized care for preterm and sick neonates. The unit follows the principles of FFC, welcoming parents as part of their neonate's care team. The intensive care rooms are open bay and do not have beds for parents. All eligible mothers who met the following criteria were recruited: (1) being the infant's birth mother; (2) having the infant participate in a randomized controlled trial (RCT) evaluating the effectiveness of non‐pharmacological pain relief methods during a heel lance procedure (ClinicalTrials.gov NCT04967118); (3) being Finnish‐speaking; and (4) being willing to participate in the study.

The purpose of the second inclusion criterion was to ensure that the mothers had at least one experience with both DMPI and SSC in relieving their newborns' pain. The RCT employed a crossover design, in which infants received DMPI and maternal SSC in a randomized order during the heel lance. In accordance with standard unit practice, infants also received oral glucose in combination with DMPI and SSC 2 min prior to the heel lance. The implementation of DMPI and SSC is described in Table 1. The mothers were recruited for the qualitative study at the same time as their babies were recruited for the RCT.

**TABLE 1:** Implementation of the digital maternal presence intervention (DMPI) and skin‐to‐skin contact (SSC).

Intervention	Description of the intervention
Digital maternal presence intervention (DMPI)	The mother's heartbeat was recorded for 20 min in a quiet room on the ward The heart sounds were transferred to a Nucu pad The Nucu pad is a multisensory device that allows infants to both hear (auditory sense) and feel (tactile sense) their mother's heartbeat The pad was placed under the infant's mattress The DMPI started 30 min before blood sampling Heart sounds were played throughout the procedure and for 15 min after it was over The sound volume was kept below 50 dB in accordance with the recommendations of the American Academy of Paediatrics (AAP) (Sibrecht et al. 2024) Mothers were allowed to be present during the use of the DMPI for pain relief, but their presence was not required
Skin‐to‐skin contact (SSC)	Skin‐to‐skin contact was initiated 30 min prior to taking the blood sample The mother was comfortably positioned slightly upright in the armchair A nurse lifted a naked, diapered infant onto her mother's bare chest. She turned the infant's face to one side, tucked its legs up, and brought its hands up near its face Skin‐to‐skin contact was maintained throughout the procedure and for 15 min afterward, or longer if the mother desired Mothers were allowed to carry out SSC in a way that felt natural to them They could talk to or stroke their infants while using the SSC

### 2.3 Data Collection

Semi‐structured interviews were chosen as the research method because they allow participants to express their personal thoughts and beliefs openly (DeJonckheere and Vaughn 2019). The first author conducted all the face‐to‐face interviews after the infant had participated in all interventions under study. Mothers could choose the most convenient time and place for the interview. All interviews were conducted at the NICU and recorded. At the beginning of each interview, maternal background information such as demographics, involvement in infant care and pain management, and number of SSC sessions was collected using a structured questionnaire. The interview themes (Table 2) were adapted from previous literature (Gates et al. 2018) and pre‐tested on three nonparticipating mothers.

**TABLE 2:** The interview guide.

Main interview question
What are mothers' views of using DMPI and SSC to relieve pain in their newborns?
1. Pain experienced by the newborn infant and the mother's previous involvement in pain relief
Could you tell me what your baby is like?
Could you tell me if your baby has had any pain during intensive care? When? What kind of pain?
Could you tell me if you were involved in your baby's pain relief before this study? How was your experience?
2. Digital maternal presence intervention for neonatal pain relief
Could you tell me how you felt about recording your heart sounds?
Could you tell me how you felt when you thought your baby could hear and feel your heartbeat during the blood sampling?
Could you describe how you thought your baby felt when he/she heard your heartbeat before, during and after the blood sampling?
3. Skin‐to‐skin contact for neonatal pain relief
Could you tell me if you have any previous experience with skin‐to‐skin contact? How was your experience?
Could you tell me how you felt about the skin‐to‐skin contact with your baby during the blood sampling? How did it make you feel?
Could you describe how you thought your baby felt to have skin‐to‐skin contact before, during and after the blood sampling?

Data collection took place over 10 months, from May 2023 to May 2024, excluding the summer months. The sample size was determined by data saturation, meaning no new codes were identified (Elo et al. 2014). Five additional interviews were conducted to ensure saturation, bringing the final sample size to 25. The interviews lasted between 14 and 30 min.

### 2.4 Data Analysis

Descriptive statistics were used to describe the background characteristics of mothers and infants. The interview data were analysed using inductive content analysis (Elo and Kyngäs 2008), guided by the research questions, which were carried out simultaneously with the data collection. During the preparation phase, the first author transcribed the interviews verbatim and reread them several times to become familiar with the data. The chosen unit of analysis was either a part of a sentence, a sentence, or a phrase. The original expressions were collected on a coding sheet and were simplified. The simplified expressions (*n* = 371) were open coded by underlining in different colours and writing notes and headings in the margins to describe the content dimensions of the data. Then, the expressions were grouped into subcategories based on similarities and differences. Each subcategory was named using words that characterise its content. The abstraction process was continued by combining subcategories (*n* = 52) into generic categories (*n* = 20) and finally into main categories (*n* = 8). The first author performed the initial categorisation, and then the entire research team evaluated it and formed the final categories.

### 2.5 Ethical Considerations

The study was conducted in accordance with the Declaration of Helsinki and the guidelines of the Finnish National Board on Research Integrity TENK (TENK 2023; World Medical Association Declaration of Helsinki 2013). Ethical approval for the study (ref: 296/2018) was obtained from the regional medical research ethics committee of the North Ostrobothnia Wellbeing Services County. Along with verbal information, the mothers received written information about the study, including the contact information of the responsible researcher. Before participating in the study, the mothers signed a document giving informed consent, including the consent for audio‐recorded interviews. Anonymity was ensured by assigning each participant an identification number. The audio recordings were stored anonymously on a password‐protected hard drive provided by the hospital's research services. The mothers were informed that they could withdraw from the study at any time.

### 2.6 Rigour and Reflexivity

The trustworthiness of this study was evaluated according to Lincoln and Guba (1985) based on credibility, dependability, conformability and transferability (Elo et al. 2014). The credibility of the study was enhanced by careful study design, a detailed description of the analysis, systematically reported results, and the diverse backgrounds of the research team (see Table S1). To improve dependability, the first author conducted the interviews according to the interview guide. The study's credibility was strengthened by using reflexivity to promote transparency and consistency throughout the analysis process. Quotations from the transcribed text were also provided to demonstrate the relationship between the data and the findings. The research findings, including demographic information about the participants, were reported as accurately as possible to enable readers to determine the transferability of the findings to a specific context. The study's trustworthiness was also enhanced by data saturation (Elo et al. 2014).

## 3 Findings

**FIGURE 1:**
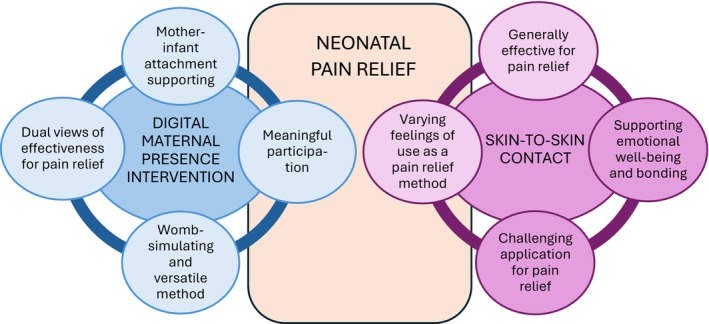
Mothers' views and experiences of digital maternal presence intervention (DMPI) and skin‐to‐skin contact (SSC) for neonatal pain relief.

A total of 25 mothers whose newborns had participated in the RCT study were interviewed. Three of the mothers had twins, but only one mother had both babies included in the study. Demographic information on mothers and newborns is shown in Table 3. Figure 1 summarizes the main categories identified in our analysis of the mothers' views with DMPI and SSC.

**TABLE 3:** Demographic information on mothers and their infants.

Mothers' backgrounds (*n* = 25)		Newborn infants' backgrounds (*n* = 26)	
**Age in years**		**Gender (%)**	
Mean ± SD	32.0 ± 6.3	Girl	35
Range	21–46	Boy	65
**Education (%)**		**Birth age in weeks of gestation**	
No vocational education	20	Mean ± SD	36 + 4 ± 3.0
Vocational education	36	Range	32 + 2–40 + 5
Polytechnic education	28	**Postnatal age in days at first intervention**	
University education	16	Mean ± SD	3.8 ± 2.8
**First‐time mothers (%)**	36	Range	1–11
Distance from home to hospital (km)		**Heel lances before the study**	
Mean ± SD	43.7 ± 49.6	Mean ± SD	12.7 ± 6.3
Range	2–160	Range	4–26
**Participation in newborn's care (%)**		**Other pain noticed by mother (%)**	
Daily	92	Prolonged pain	38
Almost daily	8	Other procedures	42
**Previous participation in pain relief (%)**		Treatments	46
Have participated	36	Psychological pain	4
Have not participated	64		
**Previous skin‐to‐skin contact before study (%)**			
Yes	58		
No	42		

### 3.1 The Use of Digital Maternal Presence Intervention for Neonatal Pain Relief

**FIGURE 2:**
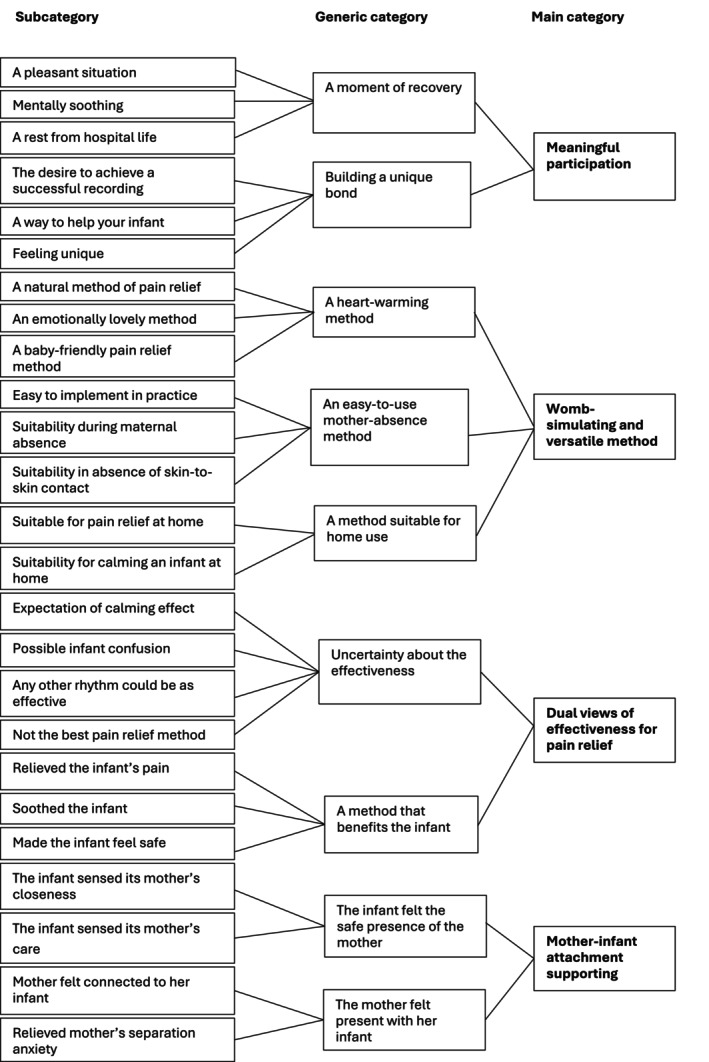
Mothers' views and experiences of the digital maternal presence intervention (DMPI).

The mothers' views of the use of DMPI in neonatal pain relief consisted of four main categories: (1) meaningful participation, (2) womb‐simulating and versatile method, (3) dual views of effectiveness for pain relief and (4) mother‐infant attachment supporting (Figure 2).

#### 3.1.1 Meaningful Participation

This main category was composed of two generic categories: (1) a moment of recovery and (2) building a unique bond.

The mothers described the recording of their heart sounds as a moment of recovery from hospital routines. Despite recovering from vaginal childbirth or a C‐section, they did not find the recording tedious or difficult. The mothers felt that all they had to do was lie down and relax. Several said the recording process was mentally soothing and offered a respite from hospital life. They had walked between the maternity ward and the NICU, unaware of how tired they were and oblivious to their own needs.

The situation itself was kind of nice. It came at a good time. I realized that I might not have rested at all, but I was able to calm myself down for about twenty minutes, and it was a really nice experience. (M015)

The mothers perceived recording their heart sounds as a meaningful way to build a unique bond with their infants, and they wanted to do it well. At the beginning of the recording, the mothers paid attention to their heart rate. They wondered if their heart rhythms were steady enough and if their heartbeats would sound different during the recording. They also wondered if the sound of the heartbeat would soothe the infant. However, soon after the recording began, the mothers felt confident that their heart rates were suitable.

In the beginning, I thought it had to be perfect somehow. But I calmed down quickly when I started to think that when he [the baby] was in my stomach, he'd heard my heartbeat and stomach rumbling for weeks. (M004)

The mothers perceived the recording of their heartbeats for their newborns as an experience that made them feel unique. They believed it was important for their babies to hear their heartbeats and that they were the only ones who could make the recording. Some reported that they didn't focus on the pleasantness of the experience but thought their newborns would soon be able to listen to their mother's heartbeats, which they believed could alleviate pain. Some mothers considered that premature infants in particular would benefit from hearing their mothers' heartbeats.

I had a feeling that I believe that it [maternal heartbeats] matters. And especially with these premature babies, when they are taken from the womb a little too early, from the familiar environment, the familiar soundscape, and so on. (M011)

#### 3.1.2 Womb‐Simulating and Versatile Method

This main category consisted of three generic categories: (1) a heart‐warming method, (2) an easy‐to‐use method in the absence of the mother and (3) a method suitable for home use.

Mothers reported that using heart sounds to relieve their newborns' pain was heartwarming. They believed it was a natural method because the infants had listened to similar sounds for nearly 9 months in the womb. The mothers believed that their infants felt good in the womb and that the recorded maternal heart sounds could recreate that feeling in the NICU.

It feels quite natural that when the baby has just been taken out of my womb, where it felt and heard all my heartbeats and stuff like that… It seems somehow logical and natural that it could actually bring her that womb feeling. It's a bit of a miserable situation now, when she's in intensive care and has all those tubes and other things. (M027)

The mothers liked the idea of relieving their newborns' pain with familiar in utero sounds. They also found it interesting that heart sounds could be recorded and played through a multisensory pad to relieve infants' pain. The mothers thought that using heart sounds for pain relief was baby‐friendly. They believed that the uterus and the mother's SSC were both safe environments for newborns. They believed that the mother's heartbeat, transmitted through multisensory pads, could create an artificial SSC environment for the newborn. Although this environment lacks the warmth and speech of the mother, it is reassuring due to the familiar sounds.

It was a wonderful, wonderful idea to use the mother's own heart sounds. And that everyone must have different sounds. Individual. They are familiar to the baby, so surely it has a different effect than if it is just anybody's heart sounds. (M024)

Mothers found that using maternal heart sounds was an easy‐to‐use method of pain relief during hospitalization when they were not present. They felt that the digital intervention was easy to implement because the same recording could be used multiple times. The recording could soothe the infant when the mother was unavailable or when the newborn could not be held in the SSC. Some mothers referred to the maternal heart sounds as a “mother helper” or a “mother substitute”. The heart sounds created a sense of closeness when mothers could not hold their newborns in the SSC.

Of course, skin‐to‐skin contact is so lovely that I would of course choose it. But in situations where I'm not able to be with the baby or to have skin‐to‐skin contact with the baby, I would want the heart sounds so that he could listen to them. (M017)

Some mothers suggested that heartbeats through a digital device could be used at home, too. They thought the heart sounds could relieve infant pain, such as stomach pain. They also speculated that the heart sounds could calm sensitive or irritable infants during tasks such as diaper changes or help them fall asleep.

When he has had the mattress [the Nucu pad ] and I have also listened to it myself, he has slept happily on it. In a situation like that, I could use the mattress at home if I wanted to put the baby to sleep somewhere, but not close to me. In a way, it would calm him down to fall asleep in his own bed. (M027)

#### 3.1.3 Dual Views of Effectiveness for Pain Relief

This main category was composed of two generic categories: (1) uncertainty about the effectiveness and (2) a method that benefits the newborn.

Many of the mothers were unsure about the effectiveness of DMPI during heel lancing. They could not judge their effectiveness because they were not present when the DMPI was used to relieve their newborns' pain during the blood sampling. However, they hoped or assumed that the heartbeats would relieve the infants' pain.

I hope it calmed the baby and made him feel safe. I didn't see the situation myself, so I don't know at all how it went. I think that if he was nervous, he would have calmed down faster anyway, because of the heartbeats. (M002)

Some mothers speculated that the heart sounds might have confused their newborns. The infants may have wondered where the sounds were coming from and where their mother's familiar smell and warmth were. However, the mothers thought that once the newborns were accustomed to the heart sounds, they would feel safe. Some mothers were unsure whether their heartbeat recordings were more effective than other sounds. One mother reported discussing with the newborn's father whether another rhythm would be as effective as the mother's heartbeat at relieving the newborn's pain. Some mothers were unsure whether their infants could identify their heartbeats. Many mothers felt that maternal heart sounds through digital devices were not the most effective method of pain relief, and they and the newborn would prefer SSC if possible. The mothers believed that physical closeness soothes newborns the most.

But on the other hand, I wonder, does the baby really recognize that there might be a little bit of their mother in it? I don't know. But if they do, I really hope it calms them down. (M021)

Most mothers reported that playing the sound of a mother's heartbeat was beneficial for their newborns during blood sampling. Some mothers believed that the heart sounds effectively alleviated their newborns' pain. They found that the heart sounds soothed their newborns and helped them sleep after the procedure. Most mothers believed that the familiar heart sounds made their newborns feel safe in the unfamiliar intensive care environment. They thought the maternal heart sounds were the only familiar and safe element for the newborns in the NICU.

The baby calmed down listening to that [maternal heart sounds], and he had the sound on for a long time in the morning, because it had been a bit of a difficult night, he hadn't really slept and had been restless. I wondered, maybe the baby liked the sound recording. (M016)

#### 3.1.4 Mother‐Infant Attachment Supporting

This main category consisted of two generic categories: (1) the infant felt the safe presence of the mother and (2) the mother felt present with her infant.

Mothers believed that their newborns sensed their safe presence and closeness when they listened to their heart sounds. The maternal heartbeat reminded the newborn of the mother, enabling the newborn to imagine her presence. One mother reported that a newborn's cognitive abilities are insufficient to understand the impact of heart sounds. However, she believed that the infant sensed her care when listening to her heart sounds.

She could have imagined that she was somewhere close to her mother, or… Who knows if she would have felt that way, that she was in her mother's arms or something. (M011)

Many mothers reported feelings connected to their newborns through the DMPI, even though they were not physically present at the hospital. They found comfort in knowing their infants could hear and feel their heartbeats during painful procedures. The mothers wanted to spend more time with their babies, but there was nowhere in the ward where they could stay overnight. The distance from home also reduced their opportunities to be with their babies. Some mothers said that using recorded heart sounds eased their separation anxiety and calmed their minds when they were apart from their newborns.

At that stage, you're still recovering and you're thinking about how you'll be able to travel such a long distance when you get home. So then, in a way, it was comforting somehow to think that you could use those sounds. (M007)

### 3.2 The Use of Skin‐to‐Skin Contact for Neonatal Pain Relief

**FIGURE 3:**
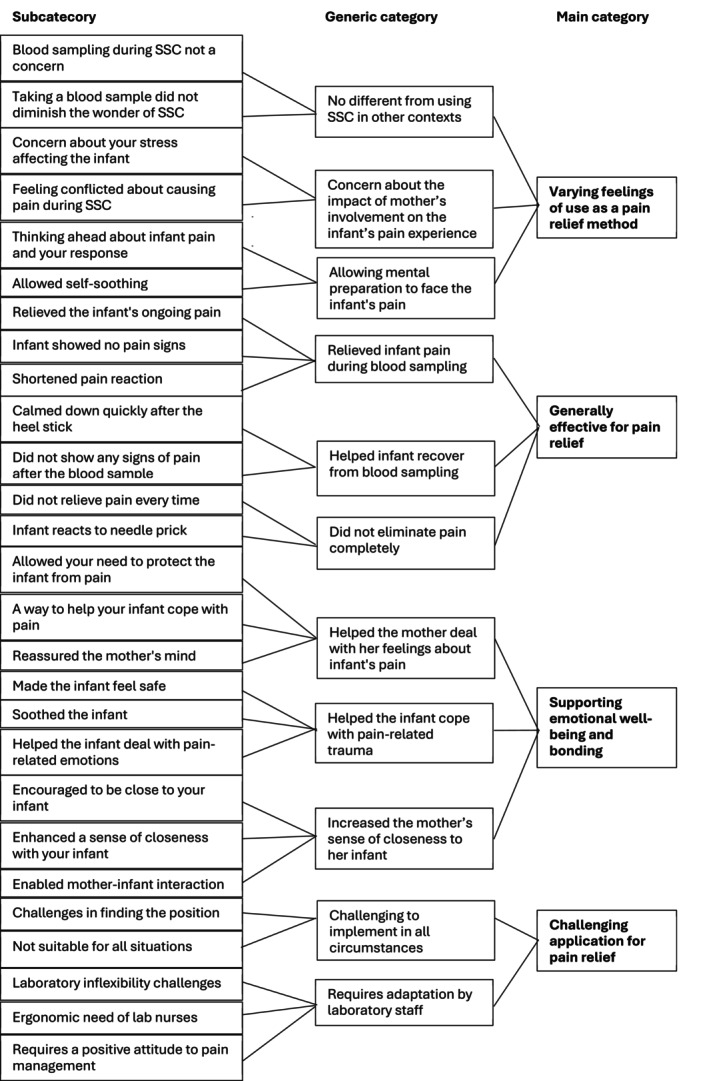
The mothers' views and experiences of the use of skin‐to‐skin contact for neonatal pain relief.

The mothers' views of the use of skin‐to‐skin contact (SSC) as a pain relief method consisted of four main categories: (1) varying feelings of use as a pain relief method, (2) generally effective for pain relief, (3) supporting emotional well‐being and bonding and (4) challenging application for pain relief method (see Figure 3).

#### 3.2.1 Varying Feelings of Use as a Pain Relief Method

This main category was composed of three generic categories: (1) no different from using SSC in other contexts, (2) concern about the impact of the mother's involvement on the infant's pain experience and (3) allowing mental preparation to face the infant's pain.

Most of the mothers had previous experience with SSC, and many felt that using it as a method of pain relief was no different from using it in other contexts. The mothers said it was wonderful to hold their newborns in SSC, and they were not nervous about having a blood sample taken during this time. They did not think about or prepare for the procedure beforehand. The mothers felt that having a blood sample taken during SSC did not diminish its wonderfulness. They only thought about how wonderful it was to hold their newborn.

I don't think I was so stressed about it now, I stroked the baby in the same way I would have done anyway. (M001)

Some mothers reported being concerned about how their involvement would impact their newborn's experience with pain. These mothers experienced emotional distress during SSC and were worried about how the blood sample would be taken, how their newborn would react to the needle, and if their newborn would sense their mother's tension. One mother felt conflicted about inflicting pain on her baby while she was sleeping peacefully in her mother's SSC. She was accustomed to the idea that when something bad happened to her child, the child would run to her lap for safety, which would make the pain go away. However, she believed that if her newborn cried, she would provide immediate comfort, which might make the painful experience less distressing.

Somehow, you were preparing yourself for the fact that the baby was about to be pricked. That's how you tried to be calm and breathe through it. And in such a way that the baby would calm down. (M015)

Some mothers reported that the SSC helped them prepare mentally to face their newborn's pain. At the beginning of the SSC, mothers prepared for their newborn's pain by thinking about how the newborn would react to pain. They wondered how painful it would be for their newborn to have a blood sample taken and whether their newborn would start crying or stay calm. Some wondered if they would be able to calm the infant if the infant expressed pain. They felt that the SSC's long duration before the newborn was pricked allowed them to self‐soothe. They verbalized the situation to themselves in their minds, but also out loud to reassure not only themselves but also the newborn. By calming themselves down, the mothers were relaxed, and some even fell asleep before the heel lance.

Somehow it came naturally to me that I wanted to put it into words. Probably for my own peace of mind, as it was. So, I said repeatedly that everything is fine, there is nothing to worry about. (M025)

#### 3.2.2 Generally Effective for Pain Relief

This main category consisted of three generic categories: (1) relieved infant pain during blood sampling, (2) helped infant recover from blood sampling and (3) did not eliminate pain completely.

Most mothers perceived that SSC relieved their infant's pain during blood sampling. One mother felt that SSC alleviated her newborn's ongoing pain before the heel lance. She described how her newborn had experienced a lot of pain in the intensive care unit due to his illness. He cried whenever he was handled or touched. However, when the newborn was lifted to the mother's breast for SSC before the blood sample was taken, he quickly calmed down and fell asleep.

That skin‐to‐skin contact took me by surprise. I expected him to cry a lot. But I don't think he cried at all. It was nice. (M026)

Most mothers perceived that SSC relieved the pain associated with the heel lance because their newborns did not react to the needle prick. They described how their newborns were completely calm and did not move or cry. In fact, they seemed to enjoy the SSC despite being pricked. Many of mothers felt that SSC eased the pain throughout the blood sampling because it kept their newborn comfortable. Some mothers compared blood sampling in bed to blood sampling with SSC. They felt that their newborns were calmer and expressed less pain during the procedure with SSC.

I remember when I was kangarooing the baby, and they took blood tests. The baby didn't move at all; he was just there. I don't think he felt any pain when they took the blood. I think kangaroo care is a good thing. (M006)

Many mothers perceived that SSC decreased the duration of their infants' pain reaction and helped them recover from the blood sampling. These mothers indicated that their infants reacted to the needle prick and/or heel pinch but calmed down fairly quickly. They thought that infants needed immediate comfort at the moment of pain to reduce it, and that SSC helped their newborns calm down and recover from the procedure. They also perceived that their infants were calm and satisfied after the procedure and that the procedure did not leave the newborns in pain.

And he continued to sleep there after the blood sample was taken, as if nothing had happened. (M002)

Several mothers found that SSC did not completely eliminate their newborn's pain. Some had more than one experience of using SSC as a method of pain relief during blood sampling. The mothers felt that the pain‐relieving effect of SSC varied. Many noticed that their newborns were expressing pain through facial expressions and movements during the blood sampling. They described their infants flinching, flexing, and grimacing quickly. Some infants expressed pain by crying a little or making noise.

I have one experience of skin‐to‐skin contact where the baby reacted very strongly. He was sampled a little bit more, maybe. And then he cried a lot. (M021)

#### 3.2.3 Supporting Emotional Wellbeing and Bonding

This main category was composed of three generic categories: (1) helped the mother deal with her feelings about the infant's pain, (2) helped the infant cope with pain‐related trauma and (3) increased the mother's sense of closeness to her infant.

The mothers described how the SSC helped them to deal with feelings related to their infant's pain. Their infant's pain triggered their protective instincts, and SSC enabled them to act on them. During blood sampling, the mothers reported talking to their infants in a reassuring way, stroking them more, shushing them, and taking better care of them. The mothers felt reassured knowing that they could use SSC to improve their newborn's well‐being and help them cope with pain. They found it reassuring to hold their infant in the SSC during the blood sampling. They said that facing their infant's pain during SSC was easier emotionally than if the infant had been in its own cot and the mother had been next to it, only able to touch the infant with her hands. One mother with a fear of needles felt that SSC was a good method. During the SSC, the mother could not see her infant being pricked with a needle or having a blood sample taken, but she could focus on her infant.

It gave me a good feeling that he was in my arms [skin‐to‐skin contact] and I could hold him. That when he was there on my skin, inside my shirt, it was really a kind of security for me too. (M004)

Several mothers believed that SSC helped newborns cope with pain‐related trauma. They thought that the infant's best place during a painful procedure was against the mother's skin. Through SSC, infants could sense familiar things: their mother's warmth, smell, sound, skin, and words. The mothers felt that SSC was like a refuge for the infants, making them feel safe and reassured. Some infants calmed down immediately, and their heart rates dropped, while others stroked their mother's breast with their hand before falling asleep. Some mothers thought that SSC with the father would also benefit the infant during blood sampling.

Maybe somehow, I felt relieved that the baby could be on my chest and I could be close to him, present. So that the baby had the most comfortable experience of it [blood sampling]. (M015)

Several mothers found that the SSC helped their infants cope with pain‐related emotions. They felt the pain experienced through the infant's mind was out of balance, but SSC allowed them to offer comfort and help their infant calm down. Some mothers found that simply holding their infant was enough, while others naturally touched their infant, talked quietly, and hummed during SSC.

This comfort came immediately [for the baby]. And the baby didn't have to suffer a lot and wait to calm down. It's quite a lot to ask of a little one in that situation to calm down on his own. (M010)

Mothers reported that SSC during blood sampling increased their sense of closeness with their newborns. Some mothers reported that their infant's poor condition had limited their involvement in care, but SSC encouraged them to be close to their infant and to be more involved in care, especially when the infant was in pain. Mothers especially felt the need to be close to and touch their infant when the infant was in pain. They felt that using SSC as a method of pain relief enabled this closeness. By holding their infant with SSC, mothers could sense that the infant was fine. The mothers also believed that SSC facilitated mother‐infant interaction.

Usually, when blood samples are taken, you put the baby in bed, and there it is. Of course, the mother may be present, touching the baby or something. But it's a much closer feeling when the baby has skin‐to‐skin contact. (M008)

#### 3.2.4 Challenging Application for Pain Relief

This main category consisted of two generic categories: (1) challenging to implement in all circumstances and (2) requires adaptation by laboratory staff.

Some mothers mentioned challenges related to implementing SSC as a pain relief method in all circumstances. They reported that their condition after a sectional delivery was an initial barrier to SSC. For some, it took several days before SSC was possible. Some also reported that their newborns' health or medical treatment prevented SSC during the first few days. Some mothers felt that finding the right position for SSC could sometimes be challenging. They were not accustomed to handling a newborn, which made holding the infant awkward and difficult.

When you can't handle the baby naturally yet, because you don't have the experience of what a good position would be. So, it's a kind of searching. Maybe the baby sensed it and got nervous about what was happening there then. (M027)

Some mothers pointed out that SSC is not suitable for all situations and can be quite challenging at times. They found that infants can undergo many painful procedures in a short period of time. They thought that SSC would not work as the main method of pain relief in the NICU because it is not a quick method that can be implemented immediately when necessary. One mother supported her infant during the insertion of an intravenous cannula. She felt that SSC or other forms of parental involvement in pain management would not have been appropriate in that situation because the cannula insertion was a very challenging procedure.

I also might not want to take the risk in that situation, that I myself might get so scared that it kind of goes wrong. Maybe I would let the doctors and nurses take care of the pain management in the case of a major procedure like that. (M025)

Several mothers pointed out that using SSC as a method of pain relief during blood sampling requires laboratory staff to adapt. They believed that the inflexibility of the laboratory made implementing SSC challenging. In a large hospital, blood sampling occurs frequently, and situations change quickly. The mothers felt that, at the moment, it was not realistic for the laboratory to be flexible enough in all situations to make SSC a feasible method of pain relief. Additionally, the mothers noted that the ergonomics of the nurses performing blood samplings were not always ideal and could hinder taking blood samples during SSC. They thought using SSC as a pain relief method required addressing the ergonomics of laboratory nurses. For example, they suggested using suitable chairs to improve posture. One mother believed that the most important aspect of implementing SSC during blood sampling was having a positive attitude towards infant pain management.

In health centers, samples are taken in such a way that the laboratory nurse is sitting in a saddle chair, so there are ways of being able to do it [during skin‐to‐skin contact]. So the baby's best interests would always be taken into account. So everyone taking blood samples understands that the baby's best interests should always come first. (M003)

## 4 Discussion

This qualitative study provided new insights into how mothers viewed using DMPI and SSC to relieve neonatal pain in a NICU. Previous quantitative studies on the use of non‐pharmacological methods for neonatal pain relief have primarily examined their effectiveness (Johnston et al. 2017) and the involvement of parents in pain management (Eissler et al. 2022). By focusing on mothers' views and experiences, this study enhances our understanding of how DMPI and SSC can support family‐centred neonatal pain management.

Mothers found DMPI for managing newborn pain to be natural and easy to use. They believed that heartbeats were useful for soothing newborns when they were absent or when the infant's treatment prevented physical contact. However, some researchers have expressed concern that digital interventions that simulate parental involvement in pain management may displace actual parental involvement in comforting and managing the newborn's pain (Ullsten et al. 2019). The results of this study did not support this concern, as all mothers considered SSC to be the best method of pain relief with respect to both mother–infant closeness and analgesic effectiveness.

The mothers expressed mixed views regarding the effectiveness of DMPI for pain relief. Using DMPI for pain relief was not standard practice at the study hospital. Therefore, the mothers' views were based on a single case in which the intervention was used in an RCT. Many mothers were unsure whether the intervention relieved their infant's pain. This uncertainty may have been related to the fact that many mothers were not present when the intervention was used. The mothers who were present during the DMPI felt that it was an effective method that comforted their infants. There is little research evidence on the effectiveness of digital parent‐driven interventions for neonatal pain management (Ullsten et al. 2024). An important methodological consideration is that in our study, both DMPI and SSC were combined with glucose, which has strong evidence of effectiveness (Bueno et al. 2013). Because glucose has well‐established analgesic properties, mothers could not reliably distinguish whether their infants' comfort resulted from the maternal intervention or from the glucose itself. This limitation applied both to mothers who were present during the procedure and those who were not. Consequently, their perceptions of effectiveness reflect the combined influence of glucose and maternal interventions rather than the isolated effects of DMPI or SSC.

In this study, SSC was used as a pain relief method in which all mothers physically participated during blood sampling. Most mothers perceived SSC as an effective method, as their infants expressed little or no pain. This finding aligns with previous research indicating that SSC is one of the most effective methods for relieving pain during minor procedures (Johnston et al. 2017). However, our study also found that some mothers experienced little to no relief from their infant's pain with SSC. Nevertheless, the mothers believed that SSC was the best method because it enabled them to immediately comfort their infants. The mothers perceived that SSC is not feasible in all situations or during all procedures, but they hoped that it could be used whenever possible. This result differs from that of a Canadian study that found some parents felt the required setup and position made SSC uncomfortable and instead preferred breastfeeding as a pain relief method (Hughes et al. 2024). It is possible that infant health status is linked to these outcomes. The Canadian study involved parents of healthy newborns undergoing routine procedures, while our study involved mothers of newborns in intensive care. Although SSC can be performed in various positions, it invariably requires direct skin‐to‐skin contact between the parents and the infant (Alberta Health Services 2017). This requirement may feel more natural in the NICU setting, where SSC is widely used as a developmentally supportive care practice, than in outpatient settings involving healthy newborns, where parents can typically hold their infants more freely.

SSC can be challenging in some situations, and our study confirmed existing evidence regarding barriers to using SSC as a pain relief method. According to the mothers in our study and previous research (Harrison 2021), organizational factors, such as the hospital's practice of taking blood samples early in the morning when parents are still asleep at home, are difficult to influence. The mothers in our study felt that increasing the use of SSC for pain relief would require changing the attitudes and work ergonomics of the laboratory nurses who take blood samples. Harrison et al. addressed the knowledge gap regarding the ergonomics of performing blood sampling while infants were held in SSC by creating a YouTube training video. However, the effectiveness of the video intervention is not yet known. (Harrison 2021).

The mothers felt that both SSC and DMPI were pain relief methods that promoted the well‐being of the newborn and mother by strengthening their bond. One of the most important factors in supporting parent‐infant closeness is the opportunity to stay overnight in the NICU (Raiskila et al. 2017). However, according to a recent study, the majority of NICUs do not allow parents to stay overnight with their newborns (Lehtonen et al. 2020). Therefore, it is important to increase the use of methods that can be implemented and that parents perceive as promoting parent‐infant bonding, such as SSC with the mother when possible and digital interventions, such as recorded maternal voices in the mother's absence.

### 4.1 Limitations

To our knowledge, this is the first study to explore mothers' views of using DMPI and SSC to relieve neonatal pain in the NICU. Understanding this phenomenon is essential for identifying future research topics, making decisions about neonatal pain management, and helping healthcare professionals understand parents' perspectives on their role in relieving their newborns' pain. However, some limitations must be recognized when interpreting the results. First, selection bias may have occurred because mothers who considered FCC and parental involvement in their newborn's pain relief important may have been more likely to participate in the study. While the participants' demographic backgrounds were diverse, it is unclear whether our findings are applicable to other countries or settings because all the participants were mothers of babies treated in a NICU in northern Finland. Nevertheless, we have presented the study findings in as much detail as possible that readers can decide whether the results are transferable to a particular context.

## 5 Conclusion and Relevance to Clinical Practice

Mothers want to be involved in managing their newborns' pain and providing comfort in the NICU. They had positive experiences using DMPI and SSC for this purpose. Although they were unsure about the analgesic effect of this type of digital intervention, the mothers felt that using heart sounds fostered a sense of closeness between infant and mother when the mother could not be present or participate in pain relief. This situation reflects the reality in many NICUs, where parental presence is often limited not only by family‐related factors but also by medical or organizational constraints that can prevent parents from being present as much as they would wish. According to the mothers, SSC is an effective, mother‐led method of relieving newborn pain. SSC also promotes the well‐being of newborns and mothers by enabling interaction during emotionally challenging situations. However, staff attitudes and practices can hinder the implementation of SSC as a method of pain relief. In line with the principles of FCC, practices should change to allow mother‐led pain management in ways that mothers prefer.

## Author Contributions

Anna‐Kaija Palomaa: Critical contributions to study design; data collection; preliminary and final analysis; writing the manuscript; reviewing and editing. Eeva Talus: Final analysis; Reviewing and editing. Sirpa Keskitalo‐Leskinen: Final analysis; reviewing and editing. Tarja Pölkki: Critical contributions to study design; final analysis; reviewing and editing. All authors meet the authorship criteria and agree with the content of the manuscript.

## Funding

This study was supported by the Alma and K. A. Snellman Foundation and the Päivikki and Sakari Sohlberg Foundation. The funders had no role in the study design; data collection, data analysis or interpretation; writing of the manuscript or the decision to submit the article for publication.

## Ethics Statement

The study received ethics approval from the Regional Medical Research Ethics Committee of the North Ostrobothnia Wellbeing Services County (approval number 296/2018). The infants of the mothers in the study participated in an RCT.

## Consent

Written informed consent was obtained from all participating mothers, including the consent for audio‐recorded interviews.

## Conflicts of Interest

The authors declare no conflicts of interest.

## Supporting information


**Appendix S1:** COREQ checklist for reporting qualitative research.

**Table S1:** Backgrounds of the research team.

## Data Availability

The data that support the findings of this study are available on request from the corresponding author. The data are not publicly available due to privacy or ethical restrictions.
